# Targeted delivery of peptide functionalized nanoparticles for ameliorating myocardial infarction

**DOI:** 10.1186/s43044-025-00644-0

**Published:** 2025-05-23

**Authors:** N Abhirami, S Sudhina, Akash Chandran, Mahesh Chandran, Janeesh Plakkal Ayyappan

**Affiliations:** 1 Translational Nanomedicine and Lifestyle Disease Research Laboratory, Department of Biochemistry, University of Kerala, Kariavattom Campus, Thiruvananthapuram, Kerala 695034 India; 2 Centre for Advanced Cancer Research, Department of Biochemistry, University of Kerala, Kariavattom Campus, Thiruvananthapuram, Kerala 695034 India; 3 Department of Nanoscience and Nanotechnology, University of Kerala, Kariavattom, Thiruvananthapuram, Kerala 695581 India; 4 Department of Biotechnology, University of Kerala, Kariavattom, Thiruvananthapuram, Kerala 695581 India

**Keywords:** Drug delivery system, MI therapeutics, Myocardial infarction, Nanoparticles, Targeted delivery, Surface modification

## Abstract

**Background:**

Myocardial infarction (MI) continues to pose a significant global healthcare burden despite advances in treatment options and their effectiveness. The incidence, prevalence, and mortality rates associated with MI are rising, emphasizing the need for improved therapeutic strategies. Traditional invasive surgical methods, aimed at recanalizing blood flow to the coronary arteries, have proven insufficient in fully addressing the complexities of MI. This ongoing challenge necessitates the exploration of novel approaches to enhance treatment efficacy and outcomes for MI patients.

**Main text:**

One promising approach is the use of nanoparticle delivery systems for targeted therapy to the infarct site. When conventional methods fail to achieve adequate permeability and retention, nanoparticle strategies offer a potential solution. Functionalizing nanoparticles is a particularly effective technique, allowing these particles to conjugate with specific ligands. These ligands possess the intrinsic ability to selectively bind to receptors that are overexpressed or uniquely present at the infarct site, thereby conferring “smartness” to the nanoparticle constructs. This review delves into the various strategies employed in nanoparticle-ligand functionalization, highlighting the versatility and potential of these approaches. It provides a detailed cross section of several ligand classes, each with unique properties and binding affinities that make them suitable for targeted delivery in the context of MI. The focus is on identifying ligands that are either unique to the infarcted myocardium or significantly upregulated during MI, ensuring precise and efficient targeting of therapeutic agents.

**Conclusion:**

In summary, while traditional surgical methods for restoring blood flow in MI patients remain important, they are not sufficient on their own. By leveraging the specificity of these ligands, nanoparticles can be directed precisely to the infarct site, enhancing the delivery and efficacy of therapeutic agents. This review underscores the need for continued research into nanoparticle-ligand functionalization strategies, aiming to improve outcomes for MI patients and reduce the global burden of this condition.

## Background

According to the latest reports, coronary artery disease (CAD) is considered as the foremost cause of mortality, leading to millions of deaths each year [1]. According to Scopus, there have been only 1702 papers published on the application of nanotechnology to heart diseases in the last decade, which indicates that nanotechnology is in its infancy when it comes to cardiovascular pathology [2]. Most of the drugs, currently available for cardiovascular therapy fail to accumulate infarct tissue, resulting in minimal concentrations and ultimately causing damage to other tissues [3]. Another major concern while dealing with MI is the negligible regeneration capacity of cardiomyocytes [4]. The need for developing new regeneration techniques is highlighted by the fact that the sole therapeutic option for advanced disease conditions is organ transplantation, which is constrained due to donor shortages.

### Main text

**Fig. 1:**
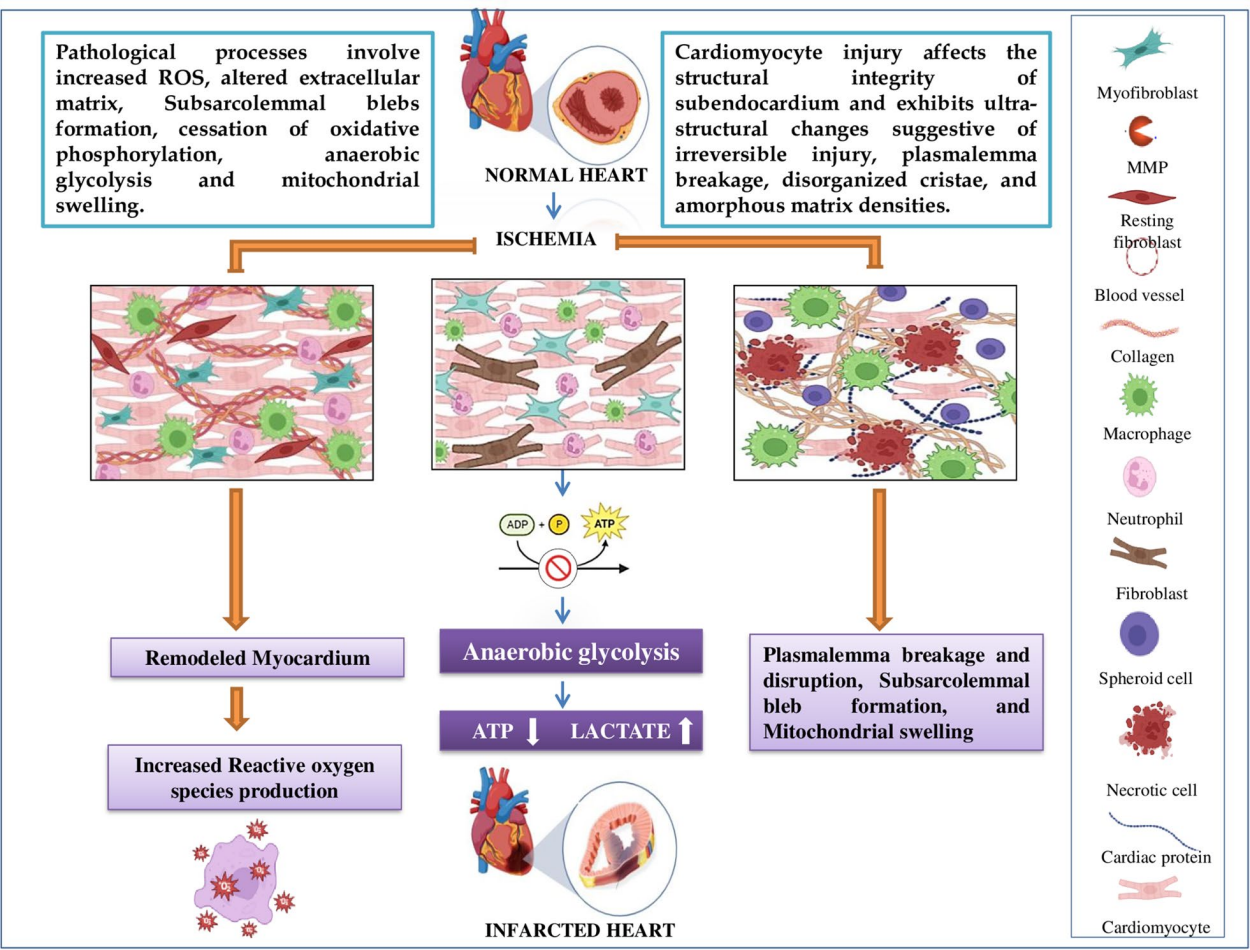
Pathological processes involve increased ROS, altered extracellular matrix, cessation of oxidative phosphorylation, anaerobic glycolysis, mitochondrial swelling, plasmalemma breakage, subsarcolemmal bleb formation, severe mitochondrial swelling, disorganized cristae, and ultra-structural evidence of amorphous matrix densities

**Fig. 2:**
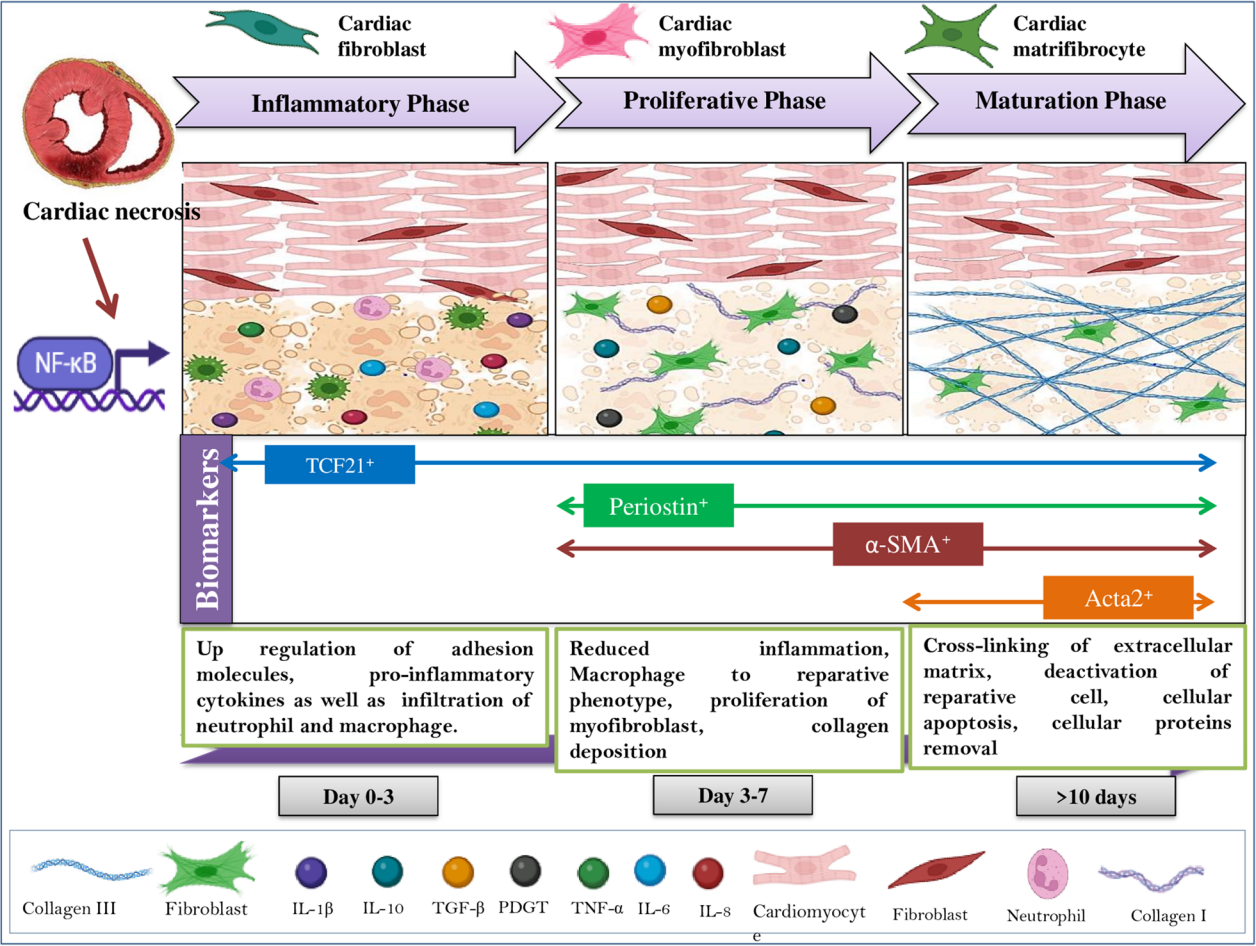
Dynamic process of cardiac remodeling and fibrosis following a myocardial infarction (MI)

MI is one of the most significant components of IHD, arising when blood flow to the myocardium is decreased or completely halted (Fig. 1). An infarction can be silent and go undetected, or it can be catastrophic, resulting in hemodynamic deterioration and sudden death [5]. MI may lead to several other complications such as heart failure, arrhythmia, cardiac arrest, or cardiogenic shock [6]. In order to restore perfusion and avoid myocardial necrosis, the current treatments for MI mostly concentrate on recanalizing the blocked coronary artery and the administration of medicines in high concentrations, which can be detrimental to other organs. Cardiac remodeling is usually followed by changes in the geometry and morphology of the myocytes. Hypertrophy and fibrosis of the left ventricular wall of the heart usually occurr during the pathogenesis of MI. During this process, there will be an increased production of chemokine and cytokines that in turn mediates the macrophage activation to clear the cell and matrix debris. This triggers the expansion of fibroblasts and secrets a collagen-based matrix network. The adult mammalian heart has an abundance of interstitial and perivascular fibroblasts that grows and helps in repair the damage, but also leads to maladaptive fibrotic remodeling. After a MI, cardiac fibroblasts undergo continuous morphological modifications that assist in governing inflammatory, restoration, and vascular responses. When the infarcted heart is repaired, the dead myocardium is eventually replaced by non-contractile collagen-based scar tissue. Resident cardiac fibroblasts in the ischemic area develop a pro-inflammatory phenotype, recruit leukocytes via cytokine secretion, and transform into myofibroblasts during the inflammatory and proliferative phases, contributing to scar formation and maintenance in heart injury. (Fig. 2).

Therefore, there is an urgent need to develop a drug delivery system (DDS) that target cardiovascular medications to the myocardium. DDSs are a category of nano-constructs that can encapsulate lipophilic as well as hydrophilic drugs thereby improving their durability and water-soluble nature, minimize their physiological clearance, and regulate their bio-distribution, inactivation, or degradation to optimize their safety and effectiveness. Because targeting means accurate accumulation of the drug in the infarct site with little impact on the neighboring healthy tissues, it is crucial for site-specific drug delivery [7]. Therefore, nanotechnology improves tissue regeneration, imaging, and drug delivery in the treatment of MI. Targeted drug delivery is made possible by nanoparticles like PLGA-PEG, liposomes, and gold nanoparticles, which also improve bioavailability and lessen side effects. Iron oxide nanoparticles enhance infarct imaging, and stem cell-loaded nanoscaffolds aid in myocardial healing. Furthermore, oxidative stress is decreased by ROS-scavenging nanoparticles (like cerium oxide), which prevent additional cardiac damage. In this review, we will explore and outline potential strategies for targeting drugs to the myocardium using nanoparticles.

### Nanoparticles tagged with proteins of interest for drug delivery

**Fig. 3:**
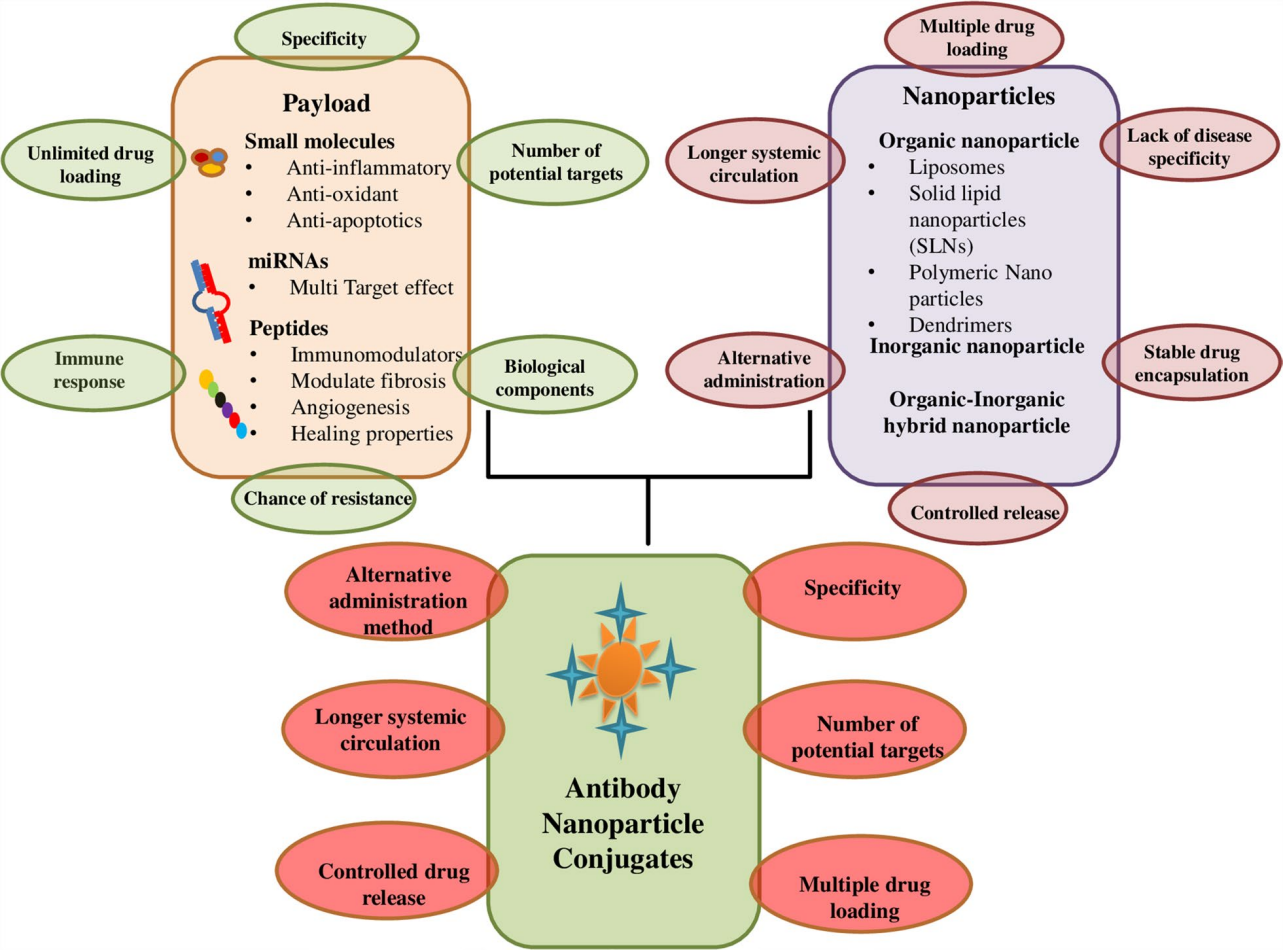
Cell internalization by active targeting nanoparticles has been suggested to improve therapeutic efficacy in comparison with non-targeted nanoparticles because the targeting ligand’s primary function is to enhance the uptake of nanoparticles into target cells. NPs promote pharmacokinetics, increased efficacy, safer and secure application, increased solubility, site-specific action and release, promote medicinal effect and boost bioavailability

The majority of recent research has focused on creating nanocarriers as prospective DDSs. The size of nanoparticles, which ranges from 1 to 100 nanometers, is much smaller than that of biological macromolecules like protein and DNA. They have the ability to interact with biological systems and can overcome biological barriers easily. The application of nano-based delivery systems include increased efficacy, improved pharmacokinetics, bioavailability, increased medicinal effect, site-specific drug release, increased solubility, and are safe and secure (Fig. 3).

**Fig. 4:**
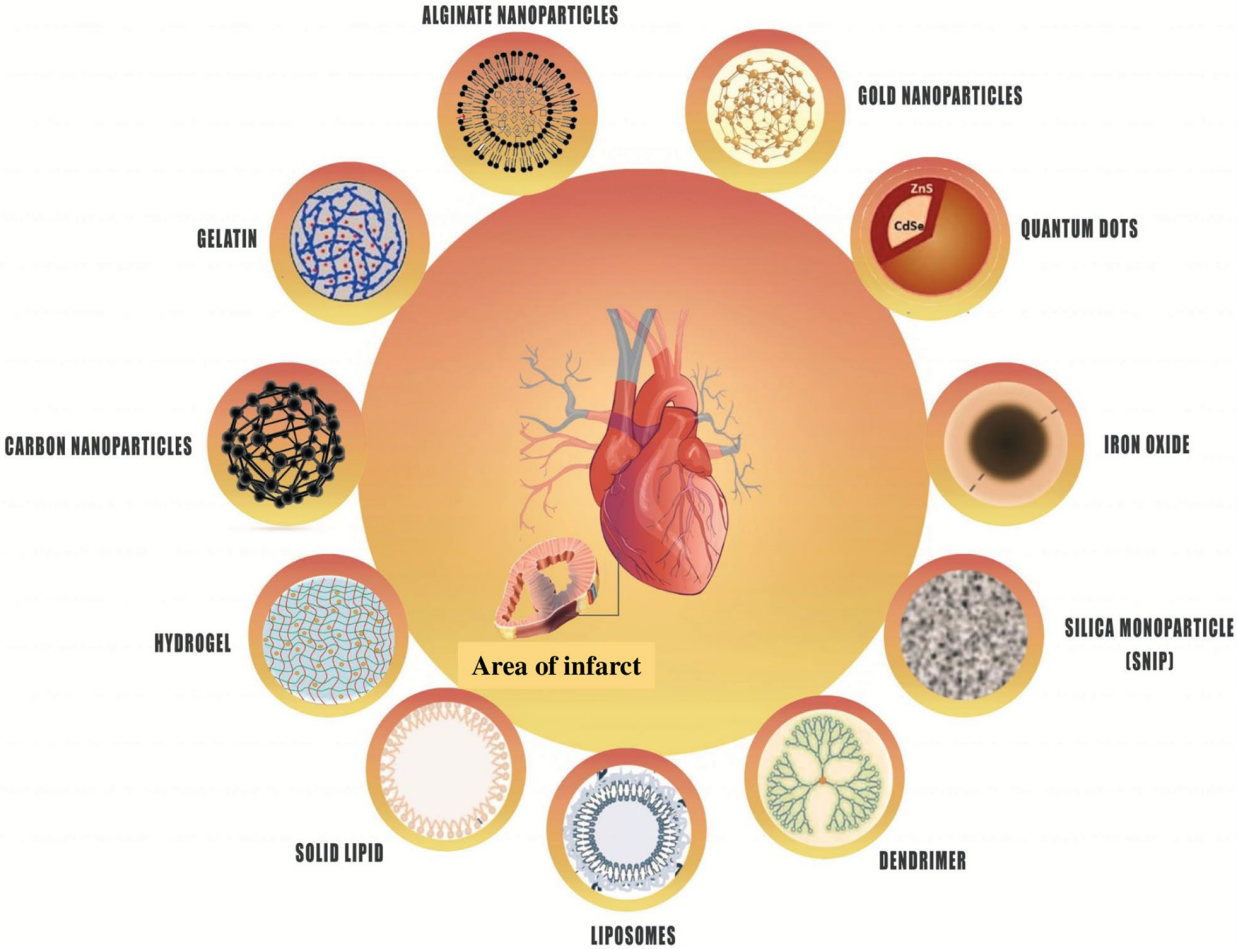
Schematic representation of different types of nanoparticles (NPs) divided into organic, hybrid and inorganic categories. Organic nanoparticles include alginate nanoparticles, liposomes, solid lipid nanoparticles, hydrogels, and dendrimers. Inorganic nanoparticles include gold nanoparticles, silver nanoparticles, iron oxide, carbon nanotubes, and silica

Nanocarriers are classified into organic and inorganic nanoparticles as well as hybrid groups [8]. Sometime different combinations of these materials are also effective (Fig. 4).

### Organic nanoparticle

#### Liposomes

**Fig. 5:**
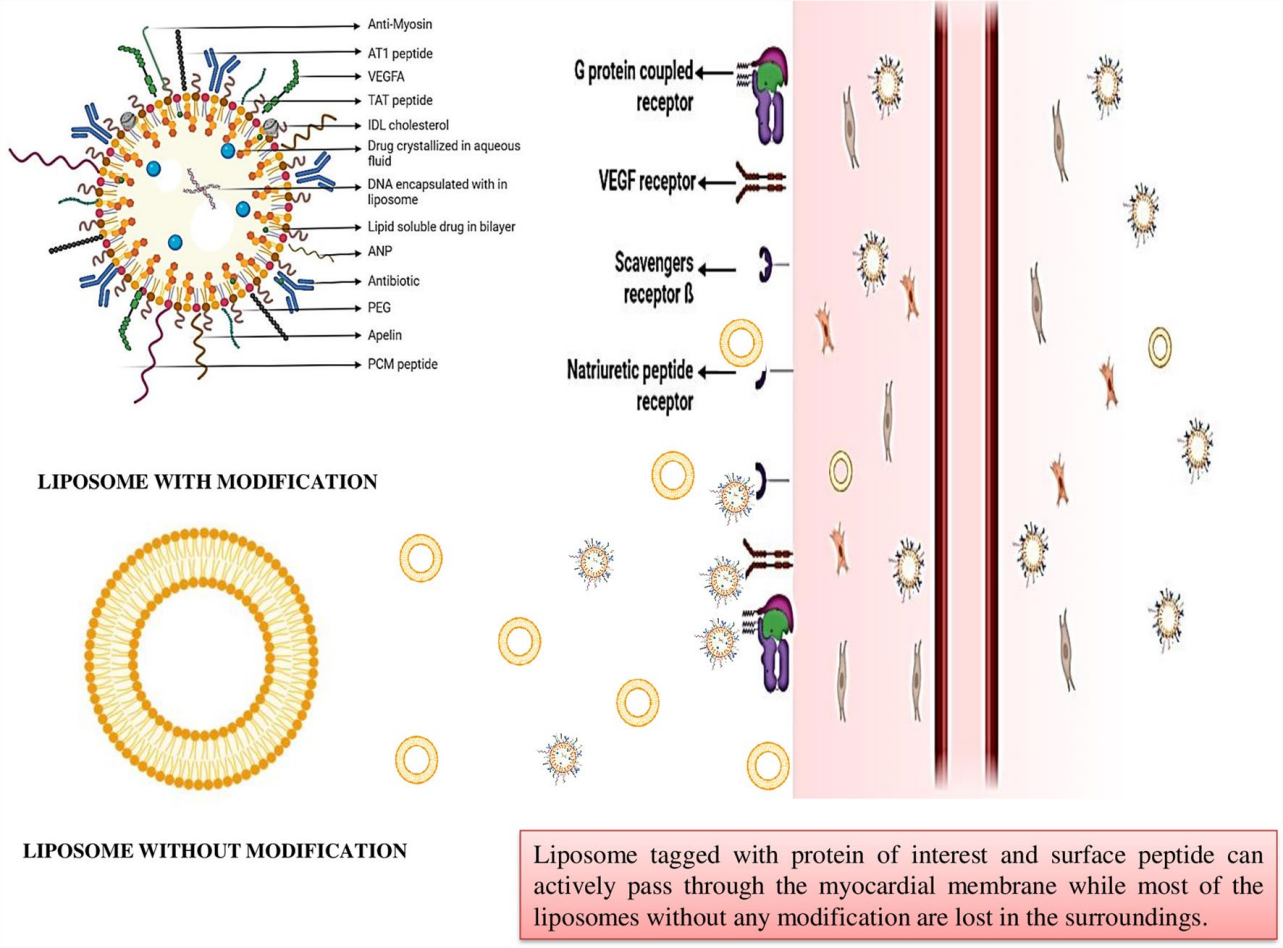
Conventional liposomes comprising phospholipids, a layer of polyethylene glycol (PEG)-specific ligand to target infarct site; which can be used for diagnosis and treatment

Lipid nanoparticles represented by liposomes are composed of a phospholipid bilayer membrane, most of the time, they take the shape of a vesicle. It has been demonstrated that the proangiogenic cytokine vascular endothelial growth factor (VEGF) encourages neovascularization in heart disease patients [9, 10]. Because VEGF plays a role in angiogenesis, its local distribution may have a major impact on the prognosis of MI by encouraging the development of neocapillaries, reducing local ischemia, and favoring the decrease in necrotic tissue. Clinical investigations have shown that therapeutic results can only be attained at high doses but not without side effects such as hypotension, retinopathy, and malignant tumor progression [11]. In 2010, Oh et al. created nanoparticles with a core made of lecithin and VEGF, and a coating made of “poly(ethylene oxide)-poly(propylene oxide)-poly(ethylene oxide) triblock copolymer” due to the short half-life, quick clearance, and requirement to maintain an optimal risk-benefit ratio of VEGF. The end product is delivered into the peri-necrotic area of the rat heart. The nanoparticle dramatically increased the quantity of capillaries in the necrotic zone with or without the gel, indicating that VEGF conjugation was effective. [12] (Figure 5).

Angiotensin II is an important component of the “renin-angiotensin-aldosterone pathway” and may be concentrated on by the nanoparticles to modulate cardiac remodeling [13]. It is known that AT1 and AT2 are at least two different types of polypeptide receptors that mediate the activity of angiotensin II [14]. The activity of AT1 receptors is enhanced in the heart during severe ischemia and after MI, and receptor-mediated endocytosis is used to internalize them when human serum albumin nanoparticles or functionalized liposomes are used to target them [15]. In the early stages of cardiovascular diseases, activation of the above pathway is compensatory, but it gradually turns maladaptive. Studies show that angiotensin II type 1 (AT1) that contain (G-G-G-G-D-R-V-Y-I-H-P-F) amino acid sequence binds to the AT1 receptor present in cardiac cells. Nanoparticles targeting ATI receptor can be prepared by conjugating ATI targeting peptide to the COO- functional group on the liposomes. During cell culture studies, there was a threefold expansion in AT1 receptors in injured ventricular cardiomyocyte, and 83% of NPs found to be concentrated in these cells [16].

Apelin 13 and apelin 36, ligands for the receptor apelin (APJ), promote coronary and peripheral vasodilation and aid in the maintenance of cardiac homeostasis. Apelin and APJ were either maintained or down-regulated in progressive hypertrophy of the left ventricle, or compensated cardiomyopathy, whereas their expression was decreased by the commencement of decompensation [17]. Apelin administration has been shown to reduce ventricular hypertrophy and improve glucose intolerance [18]. Using PEG-coated liposomes as nanocarriers for [Pyr1]-apelin-13, the insufficient cellular and *in vivo* stability of the multiple isoforms of apelin has been resolved, allowing for sustained release and minimizing muscular expansion and tissue fibrosis [19].

Another important nanoparticulate is the oleate adenosine pro-drug lipid nano-construction with atrial natriuretic peptide (ANP). To produce enhanced stability and prolonged release, adenosine was conjugated to oleate acid. The selective targeting site can be made possible with the help of atrial natriuretic peptide (ANP) tag. It is an endogenous and potent hypotensive hormone, and is found to be elevated early after MI in the infarcted myocardium [20]. ANP can be complexed with “Distearoylphosphatidylethanolaminated polyethylene glycol” (DSPE-PEG) to produce the nanoparticle transfer system. Adenosine oleate pro-drug can then be incorporated inside the core of ANP-PEG-DSPE, via the solvent evaporation method. *In vitro* experiments revealed rapid drug release and high intracellular absorption efficiency within 24 hours [21].

Dual modified liposome carriers made of cholesterol, soybean phospholipids, and DSPE-PEG are widely used as the carrier for delivering drugs to myocardium. It consists of two targeting peptides, TAT, an 11-mer peptide (YGRKKRRQRRR), and PCM, a 20-mer peptide (WLSEAGPVVTVRALRGTGSW). High-affinity PCM binds only to cardiomyocytes, whereas TAT encourages intracellular penetration by non-specific endocytosis [22].

Recombinant tissue plasminogen (rtPA) was combined by Huang et al., with PEGylated liposomes coated with the cRGD peptide for precise and regulated drug administration [23]. This membrane fusion facilitates interactions with activated platelet GPIIb/IIIa integrins and cRGD peptides on liposomes. Due to liposomal membrane destabilization, 90% of rtPA releases occured within 1 hour [24].

Pawlowski et al. developed a liposomal delivery system for each GPIIb/IIIa and P-selectin using the peptide sequences CDAEWVDVS and CGSSSGRGDSPA. The encapsulated medication is released as a result of leukocyte and active platelet breakdown by the secreted phospholipase A2 (sPLA2) enzyme [25].

KO and his group developed another multivalent targeting using anti-myosin monoclonal antibody (mAb 2G4) and TAT peptide. Cardiac myosin exposed to the extracellular surface is an indicator of MI. The receptors in ischemia cells may be recognized and bound by mAb 2GA [26].

Following ischemia events, endothelial cells exhibit an increase in P-selectin expression. To treat rats with MI, Scott and colleagues created anti-P-selectin conjugated lipid nanocarriers. HSPC, cholesterol, and DSPE-PEG were used to create anti-P-selectin complexes to nanostructures by thiol group modifications. VEGF was further incorporated to the liposomes to increase their effectiveness in vascularizing the injured myocardium [27, 28].

### Solid lipid nanoparticles (SLNs)

SLNs are lipid monolayer that surrounds a solid core that is stabilized by a surfactant. It is an alternate method of drug delivery with a greater incidence of drug entrapment for hydrophobic drugs. Dong et al. proposed another innovative solid lipid SLN system synthesis. This SLN is composed of two components: a PEG-DSPE modified with the RGD peptide, and a solid middle layer formed of a lipid excipient and soy lecithin. The v3 integrin, which is overexpressed in endothelial cells, binds to the RGD peptide. The fast drug release was noticed during the first 12 hours, and in vitro research demonstrates the buildup of RGD/PEG-PUE-SLN [29].

### Polymeric nanoparticles

One of the most efficient biodegradable copolymers is “poly (d, l-lactic acid), poly (d, l-glycolic acid” (PLGA), which is frequently utilized because of its low lethal impact, high sustainability, and biodegradability. PLGA NPs complexed with insulin-like growth factor-1 (IGF-1) provides early cardio-protection throughout the course of MI. IGF-1 has been proven to boost heart function after myocardial infarction and to induce cardiomyocyte growth. Electrostatic interaction between anionic IGF-1 and PLGA causes complexes to form into a nanovehicle [30]. Didodecyldimethylammonium bromide (DMAB) or PVA as a surfactant were used in the production of PLGA with 10 g rtPA because DMAB-stabilized NPs had a stronger affinity for fibrin clots [31]. By inhibiting opsonization, surface PEGylation of PLGA NPs enhances biocompatibility and pharmacokinetics. rtPA was enclosed in a PLGA and Fe_3_O_4_ shell, and a chitosan-cRGD peptide was grafted onto the surface to target GPIIb/IIIa [32]. This nanovehicle is made up of brush peptide-polymer amphiphiles with functional groups specific for matrix metalloproteinase. Until they enter the injured site by vascular leakage, these structures are stable in blood circulation [33]. The systemic targeting of thymosin beta 4 (T4), a 43 amino acid, to the infarcted myocardium by “cysteine-arginine-glutamic acid-lysine-alanine” (CREKA)-modified NPs was suggested to improve the therapeutic efficacy. Tβ4 promotes epicardial and coronary artery development and has been reported to be overexpressed in the infarcted heart. Tβ4 complements coronary heart functions by protecting the cardiomyocytes from apoptosis and by activating epicardial progenitor cells. Oduk et al. in 2018 loaded PLGA nanoparticles with VEGF with an average size of 113 nm.

Chitosan, an ionic hydrophilic polysaccharide, interacts with molecules that are negatively charged to form polyelectrolyte aggregates [34]. Ionic crosslinking with sodium tripolyphosphate can be used to create self-assembled chitosan NPs. Liao et al. covalently bonded trimethyl chitosan (TMC) with cRGD, allowing the nanoparticle to target GPIIb/IIIa receptors precisely [35].

### Inorganic nanoparticles

Another type of drug delivery technique is by the use of magnetic nanoparticles. Using an external high-gradient magnetic field, MNPs laden with fibrinolytic medicines can be concentrated at the thrombus [36].

Mesoporous silica nanocarriers (MSNs) are mostly used for loading of large amount of fibrinolytic drugs via both active and passive targeting processes.

Adenosine-loaded silica nanoparticles were fabricated by Galagudza and co-workers for the targeting of adenosine to the ischemic heart. When compared to free adenosine, it subsequently raises blood pressure and significantly shrinks the infarction size [37]. Silica NPs loaded with a PED and ANP peptide for active medication can be delivered to the endocardia of wounded hearts. These NPs bind to the cardiomyocytes and non-myocytes via natriuretic peptide receptors that are overexpressed in the infarcted area.

Due to multiple biological properties, including their outstanding biocompatibility, ability to easily be surface-modified with diverse functional groups, visible light extinction behavior, electrical, sensing, optical, and biochemical activities, gold nanoparticles are ideal drug carriers. Somasuntharam and co-workers synthesized 80-nm-sized gold NPs with deoxyribozyme (Dz) (Dz-AuNPs). Dz inhibits the activation of TNF-α. Dz-AuNPs have improved stability and cytocompatibility and can internalize in cardiomyocytes and macrophages. *In vivo* studies show that Dz-AuNPs reduce 50% of TNF-α level and other pro-inflammatory cytokines that trigger the oxidative stress [38, 39].

Lately, graphene nanoparticles have shown potential as their reported bio-sensing applications have shown great diversity [40]. It consists of ultra-high surface area due to the exposure of atoms on its surface. Functionalized graphene oxide (fGO) nanosheets are impregnated with methacrylated gelatin hydrogel (GelMA) conjugated with proangiogenic human VEGF plasmid DNA that are found to efficiently transfect the heart tissue, inducing favorable effects without producing cytotoxic consequences [41]

### Chemistry of nanoparticle conjugation

Chemistry of nanoparticle conjugation is shown in Table 1.

**Table 1:** Chemistry of nanoparticle conjugation

Type of Conjugation	Linkage	Stability	Diagram
Electrostatic force	Ionic interaction	Stable	
NH2/COOH	Amide bond	Stable	
Thiol/Maleimide	Thioether bond	Stable	
Thiol/Thiol	Disulfide bond	Cleaved under reducing condition	
Hydrazide/Aldehyde	Hydrazone	Acid labile	
Click Chemistry, i.e., Azide/Alkyne	Triazole ring	Stable	
Biotin/(Strept) avidin	Non- covalent Almost irreversible	Stable	

### Physical adsorption

Physical adsorption, which primarily depends on attractive forces such as hydrogen bonds, hydrophobic, van der Waals forces, and ionic interactions, is one of the basic coupling strategies [42]. Tanaka et al. established the physical adsorption of monoclonal antibodies to the outermost layer of GNPs, which have the ability to bind antibodies to chorionic gonadotropin and the subunit of human follicle stimulating hormone (HFSH) without the requirement for any pre-modification treatments. Enhanced detection was performed with a sensitivity that was approximately 1000 times higher than the traditional detection method [43]. Another method of producing gold nanoparticles by physical interactions that were coupled with immunoglobulin G (IgG) and anti-epidermal growth factor receptor (anti-EGFR) enhanced the activity of the ligand. By minimizing the isoelectric point of IgG, they synthesized bio-conjugates that were irreversible and incredibly stable [44]. Nanoparticle-bound antibodies are released by very slight pH shifts [45].

### Covalent conjugation of nanoparticles

Covalent conjugation chemistries are the most often utilized methods for modifying nanoparticles. These covalent conjugations involve active carbonyl groups. The reactions include the formation of an oxime bond when a carbonyl reacts with an alkoxyamine, or a hydrazone bond when it reacts with a hydrazide; the formation of an amide or amidine bond when a reactive amine group reacts with an activated carboxylate or an imidoester; and the formation of an amide bond or triazole ring when a reactive sulfhydryl group reacts with an azide and phosphine or an alkyne.

### Amine-Carboxyl linkage

An established method for conjugating antibodies to the surfaces of NPs is the carbodiimide coupling method [46]. During this process, the carboxyl residues on the NPs’ surface are activated, allowing them to covalently link with the -NH group of the antibodies. Despite the fact that carboxyl moieties do not naturally attach to antibodies, they can be chemically altered to do so. In the bio-conjugation technique, reactive groups like hydroxyl and amine moieties which may chemically link with the amino acids side chains on the outermost layer of the antibody are added to the nanoparticles’ surfaces. This method can be done using identified bio-conjugation techniques such as EDC/NHS, which results in the formation of reactive NHS esters. When EDC reacts with a carboxylic group, the intermediate product O-acylisourea ester is transformed into its active form. When it interacts with a primary amine, the latter produces an amide bond. For polymers having reactive carboxyl groups in their side chains, this method is highly practical [47, 48]. The DSPE-PEG-COOH system was used, and it served as the supply of the necessary carboxylic acid groups.

### Hydrazide-Aldehyde linkage

To undergo hydrazine bio-conjugation, a molecule must have a carbonyl moiety or an alpha nucleophile present, which will undergo a simple reaction to form hydrazones under mildly acidic conditions. Typically, hydrazine bio-conjugation chemistry involves the reaction of the NH group of the amine with aldehydes and ketones on biomolecules to form an imine. A covalent hydrazone link can be created when the aldehyde group directly reacts with the hydrazide through a nucleophilic addition [49]. The residual aldehyde group found in antibodies’ Fc domains has significant reactivity with substrate surfaces triggered by hydrazides, making it a possible replacement for site-specific directed coupling. Selective bio-conjugation reaction of NP-hydrazide to aldehyde-containing antibodies involves mild periodate oxidation of the antibody, synthesis of NP-hydrazide via derivatization of NP-COOH with EDC/ADH; and conjugation of NP-hydrazide with antibody-CHO via formation of hydrazone linkage.

### Thiol-Maleimide linkage

This method uses the sulfhydryl (-SH) groups of antibodies. The -SH groups on antibodies are formed by interacting with primary amines or breaking their original disulfide bonds. Additionally, this chemical group is easily generated by using sulfhydryl-addition chemicals to modify the lysine residues’ amino. The most popular one is the Traut’s reagent [50]. At neutral pH, maleimide groups react with free sulfhydryls 1000 times quicker than they do with primary amines, increasing selectivity. Following the alkylation reaction with sulfhydryl groups, a thioether bond is created. “Sulfosuccinimidyl 4-(N-maleimidomethyl)cyclohexane-1-carboxylate (sulfo-SMCC), succinimidyl 4-(N-maleimidomethyl)cyclohexane-1-carboxylate (SMCC), and NHS/maleimide heterobifunctional linkers” are the widely used maleimide crosslinking agents [51–54]. The free thiol group is used to conjugate antibodies to the carrier.

### Thiol-Thiol linkage

Conjugation of a primary and secondary thiol group forms the disulfide bond in thiol-thiol linkage. The primary group is derived from a nanocarrier, while the secondary group is derived from a ligand. Thiol groups of a ligand can be formed by reducing disulfide functionalities or by using appropriate agents such as SATA or SPDP [55, 56].

### Click chemistry linkage

High efficiency, stereospecificity, and safe by-products are characteristics of reactions of the “click chemistry” group. Maleimide-thiol conjugation or EDC/NHS coupling are the two processes used to functionalize NPs with azide or alkyne moieties. The Huisgen cycloaddition is the most common kind of “click chemistry” reaction. When azides and terminal alkynes undergo 1,3-dipolar cycloaddition using catalyst Cu (I), 1,2,3-triazole is produced. Huisgen’s cycloaddition demands the use of reagents that can tolerate biological molecules and aqueous solutions with a wide range of pH levels [57]. Hassane et al. described the efficient liposome-ligand conjugation focused on Huisgen’s cycloaddition [58]. The ligand was an azide-group-containing derivative of D-mannose.

### Antibody-conjugated nanoparticle using adaptor biomolecules

Streptavidin, avidin, NeutrAvidin, and other molecules can be conjugated to biotin to create extremely stable complexes that can embrace a variety of chemical, pH, and temperature conditions [59]. In order to create antibody conjugates, the antibody is modified with biotin and then couples with avidin-modified nanoparticles through non-covalent interactions [60]. Avidin is a glycoprotein with a high pI, but it also tends to aggregate and bind to substances other than biotin, which reduces the specificity of the conjugate and makes it less promising than streptavidin–biotin interactions [61]. Steric factors are unlikely to prevent the coupling from occurring since each streptavidin monomer has as many as four sites for biotin binding [62, 63]. Avidin-fatty acid conjugates were employed in one investigation by Fahmy et al. to incorporate the targeted ligands. Avidin was guided into the aqueous environment using polar fatty acids (palmitic acid) to improve association with the antibodies. This coupling approach is more stable and may be employed with polymers such as PLGA, which only have a few functional chemical groups accessible for conjugation. Additionally, the increased avidin incorporation density on the particles may lead to better protein production and encapsulation when compared to unmodified particles. Furthermore, Park et al. investigated how various fatty acids affected avidin’s ability to integrate nanoparticles.

## Conclusion

This review’s main objective is to demonstrate how readily the notion of targeted drug delivery using nanostructured materials may be applied to coronary artery disorders, in addition to providing an overview of how easily this concept can be applied to coronary artery diseases. Nanotechnology-based drug delivery systems are currently in a pivotal stage of preclinical trial and scientific trial. They show tremendous effects in the diagnosis and treatment of MI. The aim of this review is to put forward an appropriate idea on nanocarriers fabricated for drug delivery and their targeting sites. Newly found nanostructures may be crucial in the development of an advanced MI diagnosis and therapy in the future. The demanding situations in gene delivery like cellular uptake and pharmacokinetics could be overcome via the usage of appropriate nanocarriers.
